# Dynamic Strength Prediction of Brittle Engineering Materials via Stacked Multi-Model Ensemble Learning and Interpretability-Driven Feature Analysis

**DOI:** 10.3390/ma18133054

**Published:** 2025-06-27

**Authors:** Xin Cai, Yunmin Wang, Yihan Zhao, Liye Chen, Peiyu Wang, Zhongkang Wang, Jianguo Li

**Affiliations:** 1 Sinosteel Maanshan General Institute of Mining Research Co., Ltd., Maanshan 243000, China; xincai@csu.edu.cn (X.C.);; 2 School of Resources and Safety Engineering, Central South University, Changsha 410083, China; 3 PowerChina Zhongnan Engineering Co., Ltd., Changsha 410014, China; 4 Changsha Nonferrous Metallurgical Design and Research Institute Co., Ltd., Changsha 410007, China; 5 Zijin Mining Group Co. Ltd., Longyan 364024, China; 6 Hunan Nuclear Geological Survey Institute, Changsha 410114, China

**Keywords:** brittle engineering materials, dynamic compressive strength, stacking ensemble model, SHAP analysis, machine learning, strain rate effect

## Abstract

Accurate prediction of the dynamic compressive strength of brittle engineering materials is of significant theoretical and engineering importance for underground engineering design, safety assessment, and dynamic hazard prevention. To enhance prediction accuracy and model interpretability, this study proposes a novel framework integrating stacking ensemble learning with SHapley Additive exPlanations (SHAP) for dynamic strength prediction. Leveraging multidimensional input variables, including static strength, strain rate, P-wave velocity, bulk density, and specimen geometry parameters, we constructed six machine learning regression models: K-Nearest Neighbors (KNN), Random Forest (RF), Gradient Boosting Decision Tree (GBDT), LightGBM, XGBoost, and Multilayer Perceptron Neural Network (MLPNN). Through comparative performance evaluation, optimal base models were selected for stacking ensemble training. Results demonstrate that the proposed stacking model outperforms individual models in prediction accuracy, stability, and generalization capability. Further SHAP-based interpretability analysis reveals that strain rate dominates the prediction outcomes, with its SHAP values exhibiting a characteristic nonlinear response trend. Additionally, structural and mechanical variables such as static strength, P-wave velocity, and bulk density demonstrate significant positive contributions to model outputs. This framework provides a robust tool for intelligent prediction and mechanistic interpretation of the dynamic strength of brittle materials.

## 1. Introduction

Geotechnical engineering materials serve as the foundational elements of civil and mining infrastructure systems. Among these materials, brittle engineering substances, such as brick, concrete, and rock, exhibit distinct mechanical behaviors under dynamic loading conditions, fundamentally deviating from their quasi-static responses [1,2]. The dynamic compressive strength of brittle materials is synergistically governed by strain rate, heterogeneity, and loading path, phenomena ubiquitously observed in engineering scenarios such as blasting, impact, tunneling, and seismic events [3,4]. As a critical parameter for assessing the dynamic stability of geotechnical systems, the accurate prediction of dynamic strength directly underpins the scientific rigor of engineering design and the effectiveness of dynamic hazard mitigation strategies [5,6]. Split Hopkinson pressure bar (SHPB) experiments have been utilized to measure material dynamic strength, but the test method has the drawbacks of high cost and complexity [7]. Consequently, advancing predictive models for the dynamic strength of brittle engineering materials not only addresses the limitations of experimental methodologies but also enhances mechanistic interpretations of dynamic responses and informs practical engineering applications.

In recent years, advancements in computational technologies and artificial intelligence have facilitated the integration of machine learning into brittle solid mechanics, enabling the development of efficient, flexible, and high-precision predictive models, particularly for material strength [8,9,10,11]. For instance, Li and Tan [8] employed Support Vector Machines (SVM) to predict static compressive and shear strengths using accessible parameters such as rock density, point load strength, P-wave velocity, and weathering durability indices, demonstrating the potential of machine learning (ML) as a robust supplement to traditional experimental approaches. Similarly, Cheng et al. [11] proposed a hybrid machine learning model integrating Artificial Neural Networks (ANN) with Particle Swarm Optimization (PSO) to predict rock shear strength parameters, including internal friction angle and cohesion. Using a dataset of 199 samples across diverse lithologies, the hybrid model demonstrated superior accuracy and generalization compared to traditional and single-model approaches, confirming its effectiveness in rock parameter prediction. Beyond static strength prediction, growing attention has turned to dynamic strength, aiming to capture the rate-dependent and nonlinear behavior of rocks under high loading. For example, Wei et al. [12] employed SVM, BPNN, and RF models to investigate the effects of geometrical, physical, and mechanical properties on dynamic compressive strength. The RF model outperformed others, with strain rate identified as the dominant factor, while static strength and P-wave velocity showed lower importance. At high strain rates, specimen size had less influence on strength but a greater effect on the Dynamic Increase Factor (DIF), reflecting a staged transition in rock response mechanisms. Yang et al. [13] extended ML to dynamic strength prediction, comparing four ML models under varying strain rates and constructing a Particle Swarm Optimization-based Support Vector Regression (PSO-SVR) model. This model outperformed others in both training and testing phases, validating its capability to address nonlinear strain rate-strength relationships. Their findings highlighted strain rate as the dominant variable influencing dynamic compressive strength, reflecting the critical role of loading velocity in rock failure mechanisms. For complex environmental conditions, Lv et al. [14] proposed a hybrid model combining the Sparrow Search Algorithm (SSA) with Random Forest (RF) to predict dynamic compressive strength under freeze–thaw cycling effects. This approach demonstrated exceptional fitting and generalization performance on 216 experimental datasets, achieving a coefficient of determination (R^2^) of 0.961, which underscores the adaptability and practical value of machine learning in modeling rock dynamic behavior under multifaceted environments. Despite these advancements, most existing studies rely on single-model frameworks or minor optimizations, which struggle to capture nonlinear interactions among complex geological conditions and multivariable inputs, resulting in limited prediction stability and generalizability.

To address these research gaps, this study proposes a novel dynamic compressive strength prediction framework of brittle materials that synergistically integrates stacking ensemble learning strategies with a SHAP (SHapley Additive Explanations)-based interpretability mechanism. The proposed methodology leverages the complementary strengths of multiple high-performance base machine learning models to significantly enhance the accuracy and robustness of predictions. Concurrently, the SHAP framework is employed to conduct interpretability analysis on model outputs, elucidating both the marginal contributions of input variables to prediction outcomes and the critical factors governing dynamic rock strength from a data-driven perspective. This dual focus on predictive performance and interpretability bridges the gap between empirical modeling and mechanistic understanding, enabling the unified advancement of model efficacy and physical insight. The developed framework provides scientific support for rock dynamic response analysis and engineering design in complex geological environments, offering a systematic approach to balance computational precision with engineering applicability.

## 2. Dataset Construction and Analysis

The dataset utilized in this study was compiled from multiple published rock dynamics experiments, systematically encompassing the dynamic response characteristics of diverse lithologies under varying loading conditions. Data sources are obtained from publicly published studies, including representative works [15,16,17,18,19,20,21,22,23,24,25], which provide comprehensive geometric parameters, material properties, and dynamic mechanical test results, thereby establishing a robust data foundation for machine learning modeling. During data integration, unit standardization was first applied to raw datasets, and samples with critical missing parameters or anomalous experimental conditions were excluded based on completeness criteria. This process yielded a structured rock dynamics database containing 180 samples.

Aligned with the physical mechanisms of rock dynamic failure and existing research frameworks, seven critical physical parameters were selected as model inputs: specimen length (mm), specimen diameter (mm), grain size (mm), bulk density (kg/m^3^), P-wave velocity (m/s), strain rate (s^−1^), and static compressive strength (MPa). These features holistically characterize geometric morphology (dimensions and grain size), intrinsic material properties (density and elastic wave velocity), dynamic loading conditions (strain rate), and quasi-static mechanical performance, thereby enhancing the model’s capability to represent multi-scale mechanical behaviors of rocks. The target variable, dynamic compressive strength (MPa), quantifies the ultimate load-bearing capacity of rocks under high-velocity impact loading. Given the limitations in field data acquisition, the input features used in this study are restricted to externally measurable macroscopic parameters, excluding volumetric damage indicators such as energy-based dangerous volumes or internal failure regions. Notably, the original dataset contained 13 missing values in grain size and 1 missing value in P-wave velocity. To address this issue, a multiple imputation method [26] was employed using the IterativeImputer module from the scikit-learn library. This approach models each feature with missing values as a function of the other variables through a multivariate imputation strategy, implemented via round-robin regression. The default estimator, Bayesian Ridge Regression, was applied iteratively to generate plausible estimates by conditional distribution sampling from observed data. This procedure preserves the statistical dependencies among variables while quantifying imputation uncertainty, thereby ensuring the structural completeness and integrity of the dataset.

As summarized in Table 1, the dataset exhibits comprehensive coverage across geometric, structural, physical, and mechanical properties, ensuring robust representation for modeling rock dynamic responses. In terms of geometric parameters, the specimen length and diameter span 10–70 mm and 2.5–70 mm, respectively, with high standard deviations (15.34 and 17.50) reflecting significant dimensional heterogeneity. This variation effectively captures size effects on rock strength. Grain size ranges from 0.03 mm to 3.5 mm, encompassing fine-grained to coarse-grained structures, which facilitates the investigation of microstructure-dependent mechanical behaviors. For physical properties, bulk density varies between 2278 and 2850 kg/m^3^, while P-wave velocity covers 2437–6651 m/s. These ranges represent a spectrum of rock states, from low-density, fracture-prone media to dense, intact structures, providing critical data for modeling the coupling effects of structural integrity and wave propagation characteristics. The strain rate spans an exceptionally wide range (5 × 10^−6^ s^−1^ to 240 s^−1^), encompassing quasi-static to high-speed loading regimes. This breadth enables dynamic response analysis across diverse loading conditions. Notably, the strain rate exhibits a high standard deviation (51.8), further underscoring the diversity of loading scenarios. Static and dynamic compressive strengths display distinct differences, with mean values of 92.27 MPa and 132.84 MPa, respectively. The maximum values (212 MPa and 358 MPa) highlight the broad spectrum of material strength responses, supporting the exploration of dynamic strengthening mechanisms.

**Figure 1:**
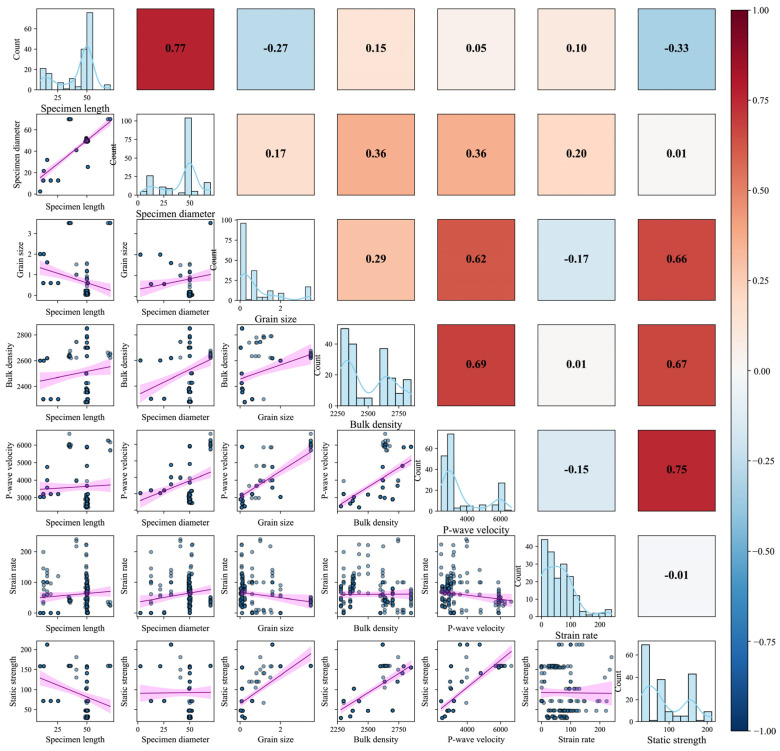
Correlation matrix of feature variables. (Colored dots represent sample points; purple bands denote locally weighted regression with 95% confidence intervals. The upper triangle shows Pearson correlation coefficients, and the diagonal displays feature distributions).

To systematically investigate the statistical interdependencies among variables, this study developed a correlation matrix of feature variables (see Figure 1). The matrix comprises three components: the upper-right quadrant displays a Pearson correlation coefficient heatmap, the lower-left quadrant illustrates scatter plots with linear regression trends for variable pairs, and the diagonal exhibits histograms superimposed with kernel density estimation (KDE) curves for individual variables. These diagonal plots visualize the marginal distributions of each feature, where the horizontal axis indicates the variable’s value range and the vertical axis represents the frequency of observations within each bin. Heatmap analysis revealed generally weak linear correlations (|r| < 0.7) among most variables, none exceeding the multicollinearity threshold (conventional cutoff: 0.8). Representative weak correlations included specimen length vs. diameter (r = 0.17) and bulk density vs. grain size (r = 0.29). Moderate positive correlations were observed between bulk density and static strength (r = 0.67), and P-wave velocity and static strength (r = 0.75), yet remained below collinearity risk levels. These findings confirm the statistical independence of input features, justifying their collective inclusion in modeling without elimination [27]. Further analysis of scatter plots identified a pronounced linear positive trend between P-wave velocity and static strength (higher velocity correlating with enhanced strength), while strain rate exhibited nonlinear oscillatory relationships with multiple parameters, theoretically validating the adoption of nonlinear regression frameworks.

**Figure 2:**
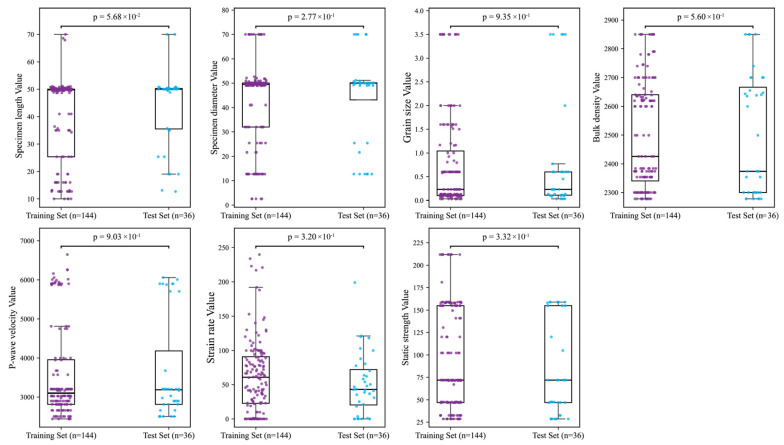
Distribution comparison between training and test sets.

To ensure model generalizability, the dataset was partitioned into training (n = 144) and test sets (n = 36) at an 8:2 ratio. Statistical homogeneity of this split was verified through Mann–Whitney U tests [28] comparing feature-wise distributions across sets, supplemented by boxplot visualizations (Figure 2). Test results indicated that all features exhibited *p*-values exceeding the 0.05 significance threshold [29], such as P-wave velocity (*p* = 0.903), strain rate (*p* = 0.320), and bulk density (*p* = 0.560), confirming distributional consistency. While the specimen length yielded a *p*-value of 0.057—slightly above the conventional threshold—it does not indicate a statistically significant difference. Moreover, given the relatively balanced central tendency and interquartile range shown in the corresponding boxplots, its marginal deviation is unlikely to adversely affect model generalization. Therefore, the overall distributional similarity is deemed acceptable, ensuring the reliability and stability of subsequent model evaluations.

## 3. Methods

### 3.1. Fundamental Principles of Stacking

**Figure 3:**
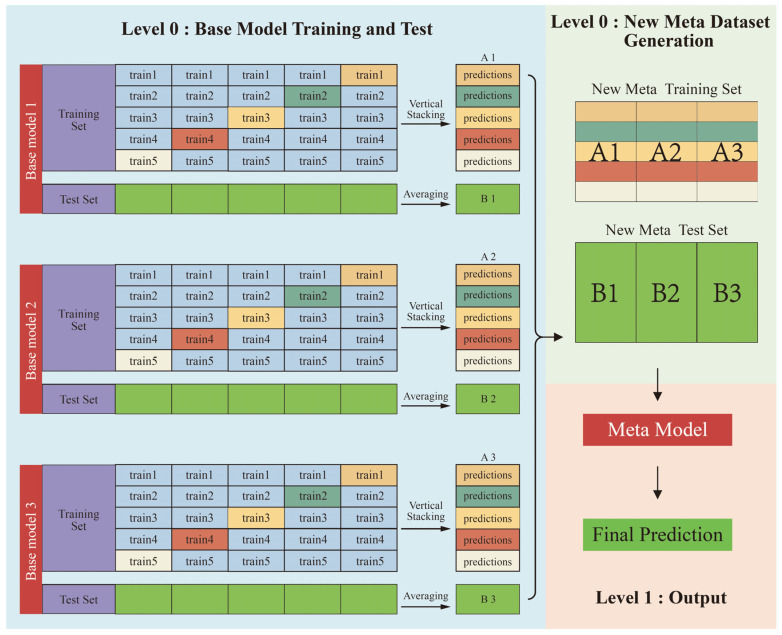
Schematic diagram of stacking ensemble technique.

Stacking (stacked generalization) is an ensemble learning technique that integrates predictions from multiple base learners (Level 0) as input features for a secondary meta-learner (Level 1), typically a simpler model, to generate final predictions [30]. The core rationale of stacking lies in leveraging a meta-learner to synthesize the diverse learning capabilities of heterogeneous base models, thereby achieving superior predictive performance compared to individual models. As illustrated in Figure 3, the training workflow of stacking comprises three key procedures:

(1) Procedure 1: Base learner training and prediction

The training data is partitioned into K subsets via K-fold cross-validation. For each fold, one subset serves as the validation set, while the remaining K-1 subsets train the base learner. The trained base learner predicts labels for both the validation set and the original test set.

(2) Procedure 2: Meta-dataset construction

Validation set predictions from all K folds are concatenated to form a meta-training dataset, which combines base learner outputs with ground-truth labels. Then, test set predictions from each fold are averaged to generate meta-test features for the meta-learner.

(3) Procedure 3: Meta-learner training and final prediction

The meta-learner is trained on the meta-training dataset to learn how to optimally combine base learner predictions. The trained meta-learner generates final predictions by synthesizing outputs from all base models.

### 3.2. Machine Learning Models

To develop a robust stacking ensemble model for rock dynamic compressive strength prediction, this study strategically selects six representative machine learning (ML) regression algorithms as candidate base learners: XGBoost, LightGBM, Random Forest (RF), Backpropagation Neural Network (BPNN), K-Nearest Neighbors (KNN), and Gradient Boosting Decision Tree (GBDT). These models collectively span diverse algorithmic paradigms, including ensemble learning, neural networks, non-parametric methods, and traditional boosting strategies, ensuring comprehensive coverage of modeling approaches and complementary predictive capabilities. Their distinct mechanisms are briefly outlined below.

#### 3.2.1. K-Nearest Neighbors (KNN)

KNN is an instance-based non-parametric regression method grounded in the principle that “similar samples yield similar responses” [31]. Unlike parametric models, KNN requires no explicit training phase, making it particularly effective for small datasets with well-defined local structures. To predict a target value for a new sample, KNN computes the Euclidean distance between the sample and all training instances [31]:

(1) $$d\left(x,x_{j}\right)=\sqrt{\sum_{k=1}^{n}{\left(x_{k}-x_{jk}\right)}^{2}}$$

where *x* is the test sample, *x_j_* the *j*-th training sample, *n* is the feature dimensionality, and *x_k_* and *x_jk_* are the *k*-th feature values of *x* and *x_j_*, respectively. The model selects the *K*-Nearest Neighbors based on these distances and averages their corresponding labels to generate the final prediction:

(2) $$\hat{y}=\frac{1}{K}\sum_{j=1}^{K}y_{i}$$

#### 3.2.2. Random Forest (RF)

Random Forest is a bagging-based ensemble algorithm comprising multiple regression trees [32]. Each tree is trained on a bootstrapped subset of the original data, with additional randomness introduced by limiting the feature subset considered at each split node. This “dual randomization” (samples and features) enhances model diversity and mitigates overfitting risks. The final prediction aggregates outputs from all constituent trees [32]:

(3) $$\hat{y}=\frac{1}{T}\sum_{t=1}^{T}f_{t}\left(x\right)$$

where *T* is the total number of trees, and *f_t_*(*x*) is the prediction from the *i*-th tree. RF excels in handling nonlinear relationships and high-dimensional data while maintaining robustness against overfitting.

#### 3.2.3. Gradient Boosting Decision Tree (GBDT)

GBDT is an iterative additive model designed to sequentially correct errors from previous iterations [33]. In each step, a new regression tree is trained to fit the residuals (i.e., differences between true and predicted values) of the current ensemble. The model’s output is expressed as follows [33]:

(4) $$\overset{\wedge }{y_{i}}=\sum_{m=1}^{M}\eta \cdot f_{m}\left(x_{i}\right)$$

where $\overset{\wedge }{y_{i}}$ is the predicted value for the *i*-th sample, *M* is the total number of trees, *f_m_* is the *m*-th tree, and *η* is the learning rate controlling the contribution of each tree. During the *m*-th iteration, pseudo-residuals for each sample are derived by minimizing the gradient of the loss function [33]:

(5) $$r_{i}^{\left(m\right)}=-\left[\frac{\partial L(y_{i},\overset{\wedge }{y_{i}})}{\partial \overset{\wedge }{y_{i}}}\right]$$

where $L(y_{i},\overset{\wedge }{y_{i}})$ is the discrepancy between true (*y_i_*) and predicted ($\overset{\wedge }{y_{i}}$) values, computed based on the gradient of the squared loss function $L={\left(y_{i}-\overset{\wedge }{y_{i}}\right)}^{2}$. Pseudo-residuals guide subsequent tree fitting, enabling GBDT to iteratively refine predictions toward the ground truth.

In this study, the GBDT model was implemented using the GradientBoostingRegressor module from scikit-learn, which employs squared loss as the default objective for regression tasks.

#### 3.2.4. Light Gradient Boosting Machine (LightGBM)

LightGBM retains the fundamental predictive principles of GBDT while optimizing for computational efficiency and scalability, particularly in large-scale, high-dimensional settings. It retains GBDT’s core architecture while incorporating three key innovations [34]: (1) Histogram-based feature splitting: Discretizes continuous features into bins to reduce computational overhead. (2) Leaf-wise growth strategy: Expands leaf nodes with maximum loss reduction instead of depth-wise growth. (3) Acceleration techniques: Gradient-based One-Side Sampling (GOSS) and Exclusive Feature Bundling (EFB) minimize data and feature redundancy.

#### 3.2.5. Extreme Gradient Boosting (XGBoost)

XGBoost further refines gradient boosting by incorporating second-order gradient information and regularization terms to enhance precision and generalization. Its objective function combines loss minimization and complexity control [35]:

(6) $$L^{\left(t\right)}=\sum_{i=1}^{n}l(y_{i},{\hat{y_{i}}}^{\left(t\right)})+\Omega (f_{t})$$

(7) $$\Omega (f_{t})=\gamma T+\frac{1}{2}\lambda {\left\|\omega \right\|}^{2}$$

where *n* is the number of samples, $l(y_{i},{\hat{y_{i}}}^{\left(t\right)})$ is the loss function, $y_{i}$ represents the true value of the *i*-th sample, ${\hat{y_{i}}}^{\left(t\right)}$ is the predicted value of the *i*-th sample after the first t decision trees are combined, $\Omega (f_{t})$ is the regularization term, introduced to control model complexity and prevent overfitting, γ is the shrinkage coefficient, T is the number of leaf nodes, λ is the regularization parameter, and ω denotes the weight of the leaf nodes.

#### 3.2.6. Multi-Layer Perceptron Neural Network (MLPNN)

MLPNN is a feedforward neural network capable of approximating arbitrary continuous functions. Its architecture includes an input layer, multiple hidden layers, and an output layer, with information propagated forward through linear transformations and nonlinear activations [36]:

(8) $$a^{\left(l\right)}=\sigma (W^{\left(l\right)}a^{\left(l-1\right)}+b^{\left(l\right)})$$

where *a*^(*l*)^ denotes the output of the *l*-th layer, *W*^(*l*)^ and *b*^(*l*)^ represent the weight matrix and bias term of that layer, respectively, and *σ*(·) is the activation function, such as ReLU or Tanh.

In this study, the MLPNN model adopted a fully connected feedforward architecture implemented via MLPRegressor in scikit-learn. It consisted of a single hidden layer with 100 neurons, as determined through Bayesian optimization. Activation functions tested included ReLU and Tanh, while the optimizer is Adam. The network was trained using the mean squared error (MSE) loss function, with a maximum of 5000 iterations to ensure convergence. The mean squared error (MSE) loss function:

(9) $$L=\frac{1}{n}\sum_{i=1}^{n}{\left(y_{i}-\overset{\wedge }{y_{i}}\right)}^{2}$$

To optimize the MLPNN performance, a Bayesian optimization strategy was employed to explore the hyperparameter space. The following parameters were tuned: activation function (relu or tanh), and L2 regularization strength alpha (ranging from 1 × 10^−5^ to 1 × 10^−2^). Five-fold cross-validation was used during the search to prevent overfitting and ensure generalization. MLPNN is well-suited for modeling complex nonlinear relationships.

### 3.3. Prediction Framework Construction

**Figure 4:**
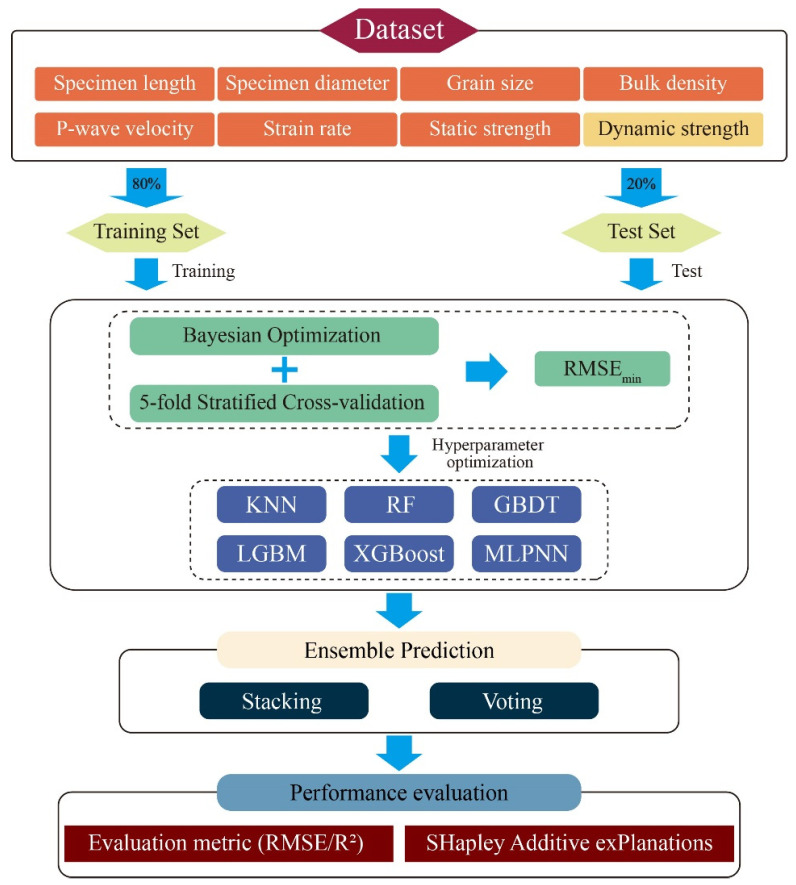
Schematic diagram of the integrated prediction framework.

To achieve high-precision prediction of rock dynamic compressive strength, this study establishes a stacking-based ensemble learning framework, as illustrated in Figure 4. The framework comprises four sequential stages: data preprocessing, base model training and optimization, ensemble modeling, and performance evaluation. All procedures were implemented using Python 3.9, with support from libraries including scikit-learn, LightGBM, XGBoost, and SHAP for model training, hyperparameter optimization, ensemble construction, and interpretability analysis. The detailed workflow is described as follows:

(1) Step 1: A dynamic rock strength database is constructed using seven representative input features: specimen length (mm), specimen diameter (mm), grain size (mm), bulk density (kg/m^3^), P-wave velocity (m/s), strain rate (s^−1^), and static compressive strength (MPa). The target variable is the dynamic compressive strength (MPa). The dataset was randomly partitioned into training and test sets at an 8:2 ratio, where the former is utilized for model training and hyperparameter tuning, while the latter serves as an independent benchmark for evaluating generalization performance.

(2) Step 2: Six regression models, including KNN, RF, GBDT, LightGBM, XGBoost, and MLPNN, are trained and optimized as candidate base learners. To enhance prediction accuracy and robustness, a Bayesian optimization strategy coupled with five-fold stratified cross-validation is employed to identify hyperparameter configurations that minimize root mean square error (RMSE) on the training set. The optimal hyperparameters corresponding to the lowest RMSE for each model are summarized in Table 2. The optimized base models are then evaluated on the test set, and top-performing candidates are selected for subsequent ensemble construction.

(3) Step 3: A stacking ensemble is implemented to integrate predictions from multiple base models, where a meta-learner synthesizes their outputs into a final prediction. For comparative validation, a Voting ensemble model is also developed.

(4) Step 4: Model performance is quantitatively assessed using RMSE and the coefficient of determination (R^2^). Additionally, SHAP (SHapley Additive Explanations) analysis is conducted to interpret feature contributions, elucidating the mechanistic influence of input variables on dynamic strength predictions.

## 4. Performance Evaluation Methodology

To holistically evaluate the accuracy and interpretability of the developed predictive model, this study employs a dual-dimensional evaluation framework: (1) quantitative assessment of numerical prediction accuracy on the test set using the root mean square error (RMSE) and Coefficient of Determination (R^2^); (2) interpretability analysis leveraging SHapley Additive exPlanations (SHAP) to quantify the contributions of input features to model predictions, thereby enhancing transparency.

### 4.1. Evaluation Metrics

In regression modeling tasks, RMSE and R^2^ serve as the most widely adopted performance metrics, capturing distinct aspects of model efficacy. RMSE quantifies the absolute deviation between predicted and ground-truth values, defined as follows [37]:

(10) $$RMSE=\sqrt{\frac{1}{n}\sum_{i=1}^{n}{\left(y_{i}-\overset{\wedge }{y_{i}}\right)}^{2}}$$

where *y_i_* denotes the true value of the *i*-th sample, $\overset{\wedge }{y_{i}}$ is the predicted value, and *n* represents the number of test samples. RMSE shares the same unit as the target variable, which facilitates the interpretation of prediction errors in practical engineering applications. A smaller RMSE value indicates that the prediction is closer to the actual value.

R^2^ measures the proportion of total variance explained by the model, expressed as follows [37]:

(11) $$R^{2}=1-\frac{\sum_{i=1}^{n}{\left(y_{i}-\overset{\wedge }{y_{i}}\right)}^{2}}{\sum_{i=1}^{n}{\left(y_{i}-\bar{y}\right)}^{2}}$$

where $\bar{y}$ denotes the mean of the target variable in the test set. The value of *R*^2^ ranges from (−∞,1], with values closer to 1 indicating better model fitting. Compared with RMSE, *R*^2^ places greater emphasis on the explanatory power of the model and is suitable for evaluating overall fitting performance. In this study, RMSE and *R*^2^ are selected as the primary performance metrics to simultaneously assess the absolute magnitude of prediction errors and the overall fitting trend, thereby providing a comprehensive evaluation of the model’s performance on the test set.

### 4.2. SHapley Additive exPlanations (SHAP)

To further elucidate the impact mechanisms of input variables on model predictions, this study employs the SHAP (SHapley Additive exPlanations) method to interpret feature contributions in the final ensemble model. Rooted in cooperative game theory, SHAP quantifies the average marginal contribution of each feature across all possible model coalitions, offering strong theoretical guarantees of local accuracy, consistency, and global additivity. Compared with alternative interpretability approaches such as LIME, SHAP provides unified local-to-global insights while preserving feature interaction effects. These characteristics are particularly beneficial in geotechnical contexts, where interactions between physical variables—such as strain rate, density, and wave velocity—are complex and interdependent. The SHAP framework is formally expressed as follows [38]:

(12) $$f\left(x\right)=\phi_{0}+\sum_{j=1}^{M}\phi_{j}$$

where *f*(*x*) denotes the model’s prediction for sample *x*, $\phi_{0}$ is the baseline value (i.e., the model output when all features are missing), and $\phi_{j}$ represents the marginal contribution of feature *j* to the current prediction. Each $\phi_{j}$ is calculated according to the SHapley value formula:

(13) $$\phi_{j}=\sum_{S\subseteq F\backslash \left\{j\right\}}\frac{\left|S\right|!\left(\left|F\right|-\left|S\right|-1\right)!}{\left|F\right|!}\left[f_{S\cup \left\{j\right\}}\left(x_{S\cup \left\{j\right\}}\right)-f_{S}\left(x_{S}\right)\right]$$

where *F* denotes the full set of features, *S* represents a subset of features, *f_S_* refers to the model’s prediction using only the feature subset *S*, and the expression in parentheses captures the marginal improvement in model output when feature *j* is added to the subset. This method traverses all possible feature combinations to compute the average contribution of each feature across all permutations, ensuring fairness and consistency in feature importance ranking.

In this work, SHAP is applied to analyze the relative contributions and directional effects of input features (e.g., P-wave velocity, bulk density, and static strength) in predicting rock dynamic strength. This approach elucidates the model’s learning mechanisms and aligns its predictions with engineering principles, thereby enhancing the model’s credibility in scientific research and engineering applications.

## 5. Results and Discussion

### 5.1. Comparative Analysis of Model Performance

**Figure 5:**
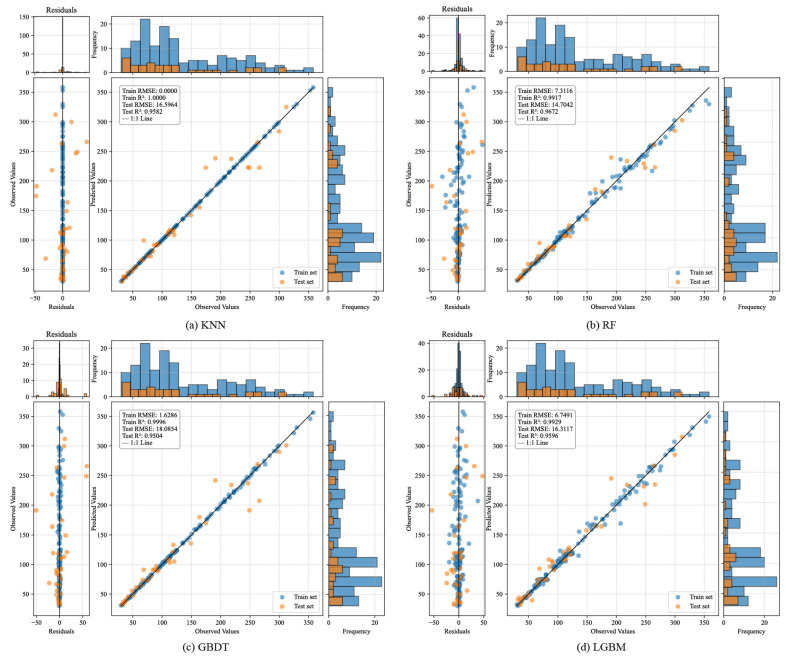

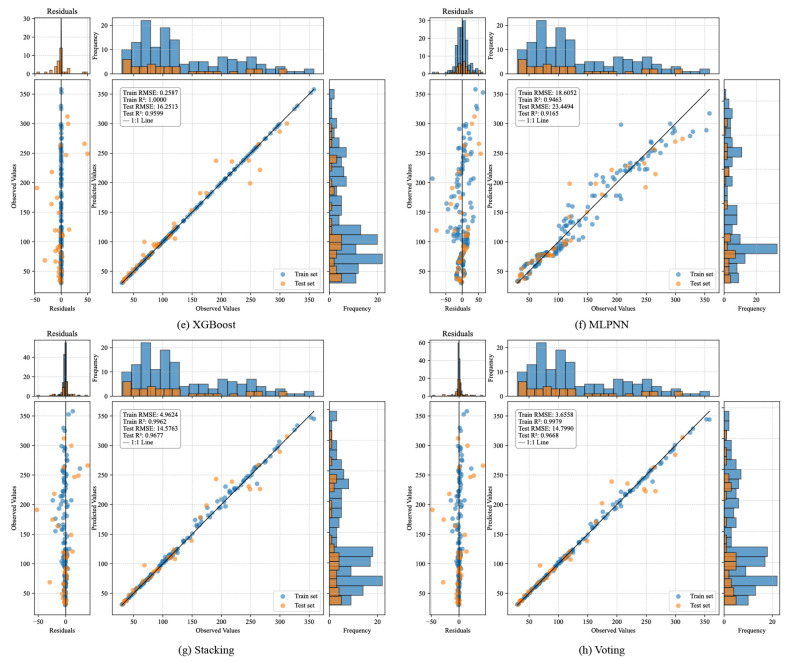
Comparative visualization of prediction performance for regression models on the test set: (**a**) KNN; (**b**) RF; (**c**) GBDT; (**d**) LGBM; (**e**) XGBoost; (**f**) MLPNN; (**g**) Stacking; (**h**) Voting (Each subplot comprises three components: Left: residual plots to observe the distribution characteristics of prediction errors. Middle: scatter plots of predicted versus true values to assess fitting trends. Right: frequency histograms of predicted and actual values to evaluate distribution consistency in the output space).

**Figure 6:**
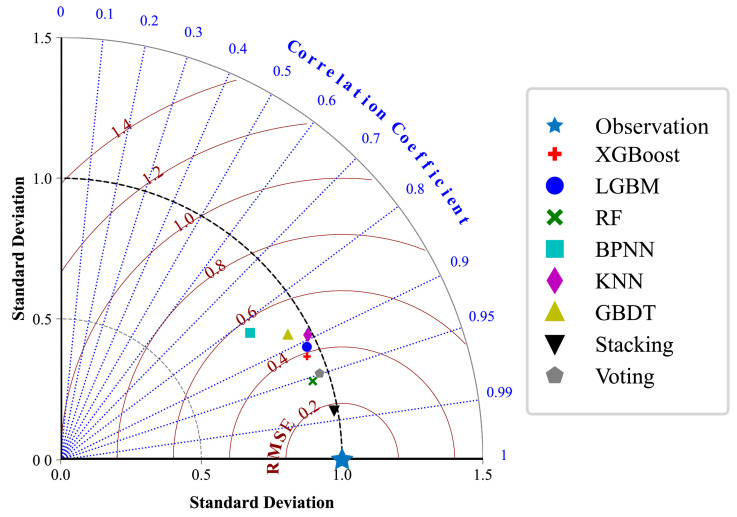
Comprehensive comparison of predictive performance across models.

To comprehensively evaluate the performance of regression models in predicting rock dynamic compressive strength, this study visualizes the residual distributions, prediction fitting effects, and output value distributions of eight regression models based on test set predictions (see Figure 5). Additionally, a standardized Taylor diagram (Figure 6) is constructed to provide a multi-metric comparative analysis across models.

Figure 5 illustrates the fitting performance of KNN, RF, GBDT, LGBM, XGBoost, MLPNN, stacking, and Voting models on both training and test sets. From the residual plots, the stacking model exhibits the most concentrated residual distribution on the test set, with significantly smaller error fluctuations compared to other models. The central portion of the residuals aligns well with a near-normal distribution, particularly within the 100–200 MPa range where most samples are concentrated. This indicates the elimination of systemic bias and superior robustness. In contrast, RF and LGBM show pronounced prediction deviations in high-strength regions (>250 MPa), accompanied by higher residual variability. MLPNN and KNN display dispersed prediction errors in low-strength intervals (<100 MPa), suggesting underfitting issues. The scatter plots further corroborate the superiority of the stacking model. Test set samples (orange points) cluster tightly around the ideal fitting line (1:1 line) across the entire strength range, demonstrating robust predictive capabilities in all intervals. Nevertheless, it is acknowledged that the residual distribution is not perfectly Gaussian, especially at the extremes. Slight right-skewness can be observed in the high-strength region (>250 MPa), and some outliers are present in the low-strength region (<100 MPa). These deviations reflect the intrinsic heterogeneity of rock dynamic behavior under extreme conditions and do not substantially affect the model’s performance in the majority region, where prediction accuracy remains high. The histograms reveal a high degree of overlap between the predicted and actual value distributions for the stacking model, particularly within the primary density range (100–200 MPa). This alignment underscores the model’s ability to capture the inherent data structure. In contrast, discrepancies in distribution shapes are evident for other models, reflecting limitations in their generalization capacity.

Figure 6 presents a normalized Taylor diagram for the visual comparison of model performance across multiple evaluation dimensions, including standard deviation, concordance correlation coefficient (CCC), and root mean square error (RMSE). Models closer to the reference point (marked by a star symbol, representing true observations) indicate predictions that align more closely with actual values. As illustrated in the figure, the stacking model is positioned nearest to the reference point, demonstrating a correlation coefficient approaching 1, a standard deviation consistent with the true values, and the lowest RMSE value, thereby exhibiting the overall optimal predictive performance. The Random Forest (RF) and Voting models rank second, showing favorable correlation but slightly higher deviations. In contrast, the K-Nearest Neighbors (KNN), Gradient Boosting Decision Tree (GBDT), and XGBoost models underperform in either accuracy or stability, particularly with significant deviations in CCC, suggesting potential overfitting or variability in these models. Notably, while the Voting ensemble integrates multiple base models, its simplistic weighted averaging mechanism fails to fully capture inter-model interactions, resulting in inferior performance compared to stacking. By contrast, the stacking framework employs a meta-learner to iteratively refine predictions by synthesizing outputs from base learners. This approach effectively balances nonlinear representation and overfitting resistance, achieving superior equilibrium between precision and robustness.

### 5.2. Model Interpretation

#### 5.2.1. Feature Importance and Attribution Across Models

To further enhance model interpretability and elucidate the mechanistic roles of input variables in predicting dynamic compressive strength, this study employs the SHAP (SHapley Additive exPlanations) method, grounded in game-theoretic principles, to analyze feature contributions across both global and local dimensions for the stacking ensemble model and its constituent base models (e.g., RF and KNN).

**Figure 7:**
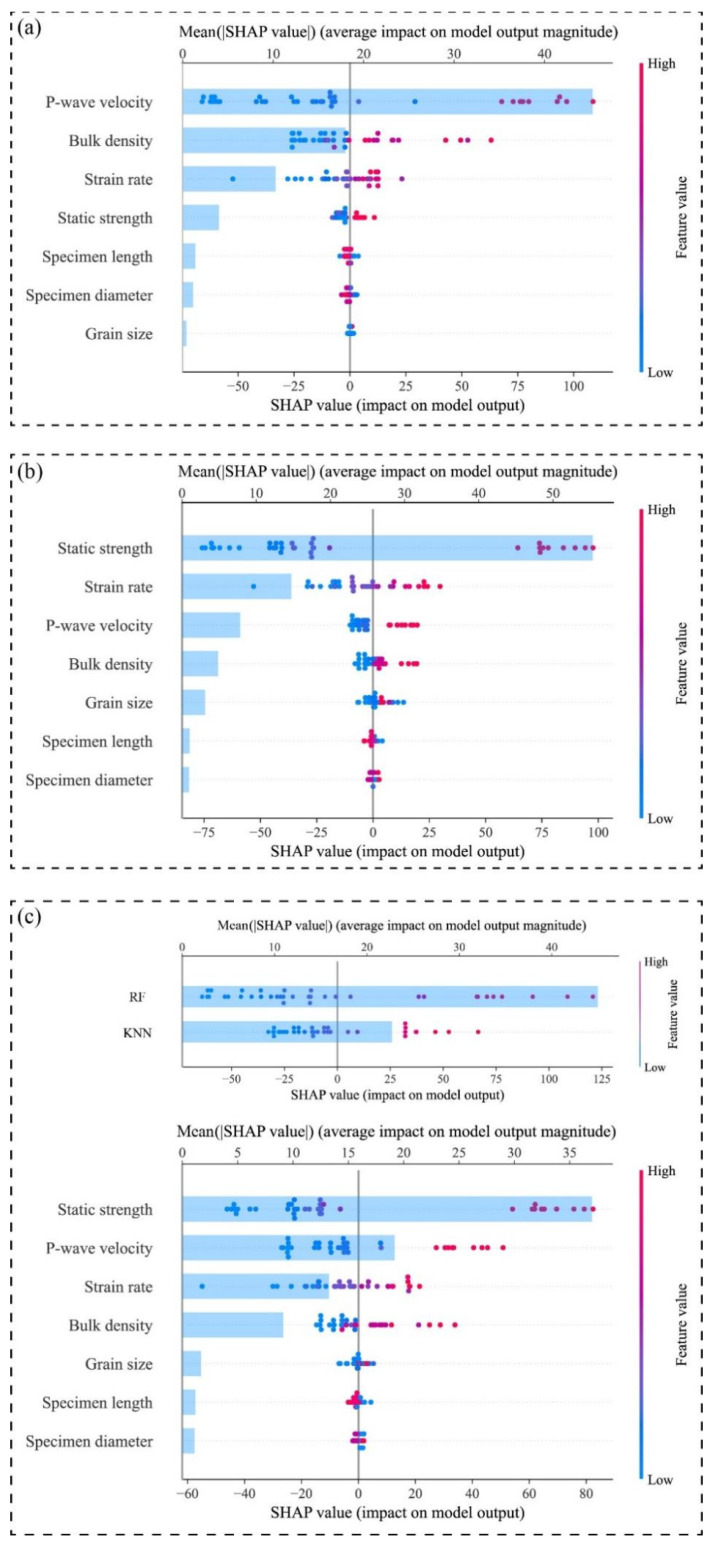
Comparative analysis of global feature contributions across models for dynamic compressive strength prediction: (**a**) KNN; (**b**) RF; (**c**) meta-model and stacking ensemble model.

Figure 7 presents a comparative SHAP analysis of feature importance for the KNN (a), RF (b), and stacking (c) models in predicting dynamic compressive strength. In each plot, the horizontal axis shows the SHAP value, reflecting both the direction and magnitude of a feature’s impact on predictions. Dots represent individual samples, with color indicating feature value. The light-blue bars denote the mean absolute SHAP value, representing overall feature importance. As shown in Figure 7a, in the KNN model, P-wave velocity and bulk density emerge as the most dominant variables. Given that KNN inherently relies on “distance-based similarity” between samples, it prioritizes variables with strong discriminative power in the sample space. P-wave velocity and bulk density, as key indicators of rock compactness and structural integrity, are strongly associated with dense, low-porosity, and low-fracture rock matrices, thereby serving as critical discriminators in KNN’s neighborhood-based predictions. Additionally, the strain rate exhibits moderate importance, suggesting that KNN partially captures the regulatory effect of loading velocity on dynamic strength, albeit with limited capacity to discern complex variable interactions.

In contrast, the RF model (Figure 7b) demonstrates a stronger reliance on discriminative features. Static compressive strength and strain rate dominate its feature importance rankings, aligning with RF’s tree-based architecture that prioritizes splitting rules for maximal class separation. Static strength, as a direct mechanical property, provides clear decision boundaries during feature partitioning, while strain rate reflects the nonlinear modulation of loading rate on strength enhancement. Compared to KNN, RF’s reduced sensitivity to P-wave velocity and bulk density highlights its preference for numerically stable, high-discriminative attributes, albeit at the cost of underrepresenting structural variables’ physical contributions.

The SHAP analysis of both the meta-model and the stacking ensemble model is presented in Figure 7c, which integrates predictions from both base models and exhibits a “balanced advantage” in SHAP analysis. It assigns high SHAP values to four critical variables: static strength, strain rate, P-wave velocity, and bulk density. This outcome indicates that the meta-model inherits RF’s sensitivity to material constitutive potential while retaining KNN’s capacity for structural feature recognition. Such multi-channel information fusion enables the stacking model to establish a physically consistent and robust mapping relationship among stress-strain paths, medium structural characteristics, and loading conditions, significantly enhancing its capability to model complex rock dynamic responses.

#### 5.2.2. Influence Patterns and Nonlinear Responses of Key Predictors

**Figure 8:**
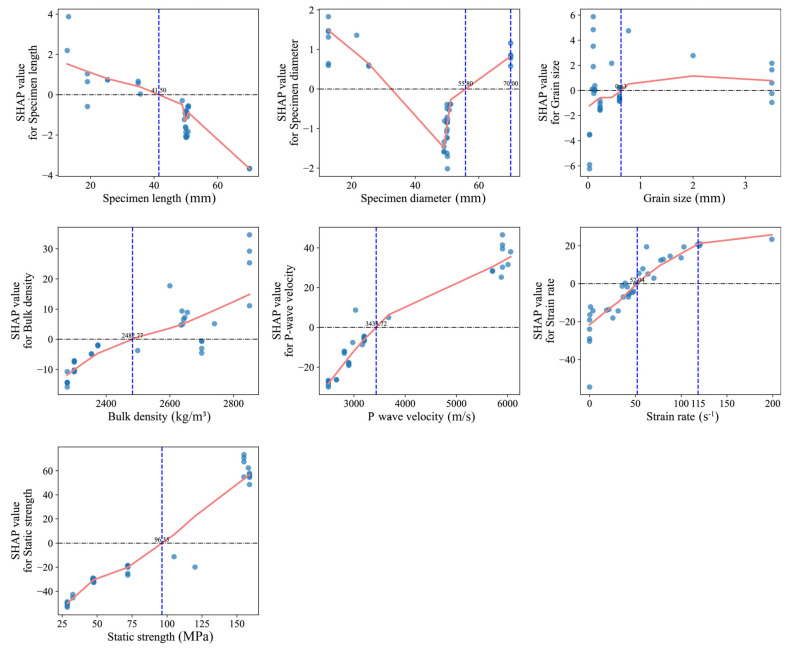
SHAP dependence plots of input variables for the stacking model output.

Figure 8 presents the SHAP dependence plots for input variables in the stacking ensemble model, illustrating the marginal effects of varying feature values on the predicted output (rock dynamic compressive strength). The plots reveal significant nonlinear relationships between multiple variables and model predictions, demonstrating the model’s capability to capture and fit complex rock mechanical behaviors. To further quantify the model’s sensitivity to key features, both global and local SHAP-based analyses were performed. These visualizations provide insight into how individual predictors influence the model’s output under varying conditions. Notably, the dependence of strain rate exhibits a critical nonlinear trend characterized by a “negative-to-positive transition followed by gradual saturation” as strain rate increases. Specifically, in the low-strain-rate regime (<50 s^−1^), SHAP values are markedly negative, indicating an inhibitory effect of strain rate on predicted strength. Within the intermediate strain rate range (50–100 s^−1^), SHAP values surge rapidly, reflecting heightened sensitivity of strength predictions to strain rate variations. At higher strain rates (>100 s^−1^), SHAP values stabilize with diminishing marginal effects, signifying a transitional mechanism from inertia-dominated enhancement to fragmentation-controlled failure.

Furthermore, the static compressive strength exhibits a stable positive correlation with SHAP values, with its marginal contribution significantly increasing within the 70–120 MPa range. This observation indicates that static strength serves not only as a core indicator of rock bearing capacity under static loading but also demonstrates substantial explanatory power under moderate dynamic loading rates, thereby validating the existence of a mapping relationship between static and dynamic strength regimes. For structural indicators, the SHAP dependence curves of P-wave velocity and bulk density both exhibit monotonic upward trends. Notably, the marginal contribution of P-wave velocity intensifies markedly beyond 3400 m/s, while bulk density displays a nonlinear surge near 2600 kg/m^3^. These patterns reflect the effectiveness of these parameters in characterizing rock compactness and structural integrity. Specifically, higher P-wave velocities signify enhanced structural continuity and reduced fracture density, which collectively improve resistance to impact-induced failure. Concurrently, increased bulk density correlates with reduced porosity and more uniform energy propagation, reinforcing material rigidity under dynamic loading. Geometric variables (specimen length, diameter, and grain size) exhibit relatively lower SHAP contributions but retain discernible patterns. For instance, a distinct inflection in specimen diameter near 55 mm may be attributed to size effects, where larger specimens are more prone to stress concentration and boundary disturbances during loading, thereby modulating macroscopic strength expression. Specimen length exhibits a downward SHAP trend, with a shift from positive to negative contributions near 41.5 mm. This suggests that shorter specimens may elevate predicted dynamic strength due to stronger boundary reflections and shorter energy dissipation paths. In contrast, longer specimens are more prone to stress wave attenuation and dispersion, leading to reduced strength contributions. Grain size demonstrates subdued marginal influence, suggesting its role may be confined to indirect regulation of crack propagation pathways in specific lithologies. Taken together, these SHAP dependence patterns confirm the model’s sensitivity to mechanically meaningful features such as strain rate, static strength, and P-wave velocity. This further demonstrates that the stacking ensemble model effectively captures key physical mechanisms governing rock dynamic response under high-strain-rate loading. These insights enhance the interpretability of the model’s internal decision-making processes and physically corroborate the multi-factor coupling mechanisms governing rock strength evolution. They also provide a theoretical foundation for subsequent engineering interpretations and parameter optimization.

**Figure 9:**
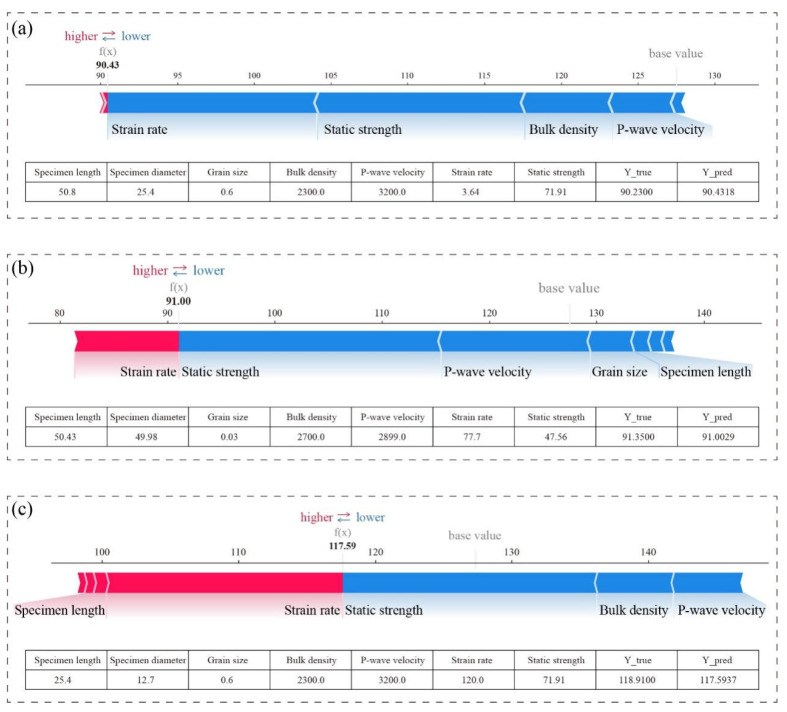
SHAP force plot analysis for samples across strain rate intervals: (**a**) low strain rate; (**b**) medium strain rate; (**c**) high strain rate.

To further investigate the significance of strain rate on dynamic strength prediction and its marginal response characteristics, this study categorizes strain rates into three representative intervals based on the aforementioned SHAP dependence analysis: suppressive response zone (<50 s^−1^), sharp-growth zone (50–115 s^−1^), and gentle zone (>115 s^−1^). Within each sub-interval, a representative sample is selected, and SHAP force plots are generated to elucidate the marginal contribution differences of input features (see Figure 9).

Each force plot visualizes how individual feature contributions (positive in red, negative in blue) shift the prediction from a base value (the model’s average output) toward the final predicted value. The longer the bar, the greater the feature’s influence on the prediction. The bottom table lists the original input values of each feature along with the predicted and actual dynamic strength. In the low-strain-rate sample (strain rate = 3.64 s^−1^, Figure 9a), the SHAP value for strain rate is negative, indicating its inhibitory effect on dynamic strength prediction within this regime. Analysis of supplementary input features suggests that at low loading rates, crack propagation and coalescence dominate rock failure, leading to increased energy dissipation pathways and reduced macroscopic strength. Consequently, the model predicts lower dynamic strength values, demonstrating alignment between physical processes and model outputs. For the medium-strain-rate sample (strain rate = 77.7 s^−1^, Figure 9b), the SHAP value for strain rate transitions to positive with a marked increase in magnitude, signifying its dominant positive contribution to model predictions in this interval. During this phase, the rock enters an inertia-enhanced response regime where crack nucleation is suppressed. Stress wave-induced energy accumulation occurs rapidly, resulting in significantly higher dynamic bearing capacity, as reflected in the model’s elevated strength predictions. In the high-strain-rate sample (strain rate = 103.0 s^−1^, Figure 9c), the SHAP value for strain rate continues to rise but exhibits a decelerating growth trend compared to the medium-rate interval. Concurrently, the relative contributions of other features increase. This pattern suggests that while the model recognizes strain rate’s critical role in strength enhancement at high loading rates, it also captures the physical phenomenon of dynamic strength entering a gentle zone. Specifically, as strain rate further increases, the rock failure mechanism shifts from crack extension dominance to pulverization, leading to diminishing marginal strength gains per unit strain rate increment. These findings validate the applicability and reliability of the proposed stacking ensemble model in addressing complex rock mechanics challenges.

## 6. Conclusions

This study presents a predictive framework that integrates a stacking ensemble learning strategy with SHapley Additive exPlanations (SHAP) interpretability mechanisms, aiming to improve the accuracy of dynamic compressive strength prediction for brittle engineering materials under dynamic loading conditions and to advance the mechanistic understanding of key controlling factors. The principal findings are summarized as follows:

(1) The proposed stacking framework achieves superior predictive performance and robustness compared to traditional single-model approaches. By leveraging the complementary strengths of diverse base learners—such as Random Forest (RF) for capturing material constitutive characteristics and K-Nearest Neighbors (KNN) for sensitivity to geometric and structural features—the ensemble balances nonlinear learning capability with physical consistency. Specifically, it achieved a test R^2^ of 0.9677 and RMSE of 14.57 MPa, outperforming the best individual model (XGBoost: R^2^ = 0.9613, RMSE = 15.42 MPa), thus demonstrating enhanced generalization and reduced prediction error.

(2) Global SHAP analysis identifies strain rate, P-wave velocity, bulk density, and static strength as the dominant predictors influencing model outputs. Strain rate exhibits the most significant marginal impact, with SHAP dependence plots revealing a characteristic “suppression–enhancement–saturation” nonlinear trend. Local force plots further indicate that strain rate initially acts as a suppressive factor at low levels (<50 s^−1^), becomes a strong enhancer within the intermediate range (50–115 s^−1^), and finally plateaus with diminishing marginal effects at high rates (>115 s^−1^). This trend reflects the model’s ability to capture inertia-driven strengthening and saturation mechanisms in rock dynamic failure.

(3) Static compressive strength demonstrates a robust positive correlation with dynamic strength, validating its role as a critical prior input variable. Structural indicators, such as P-wave velocity and bulk density, exhibit monotonically increasing SHAP values, confirming the pivotal role of material compactness and integrity in dynamic resistance. While geometric variables (e.g., specimen dimensions and grain size) show weaker overall contributions, localized influences within specific parameter ranges highlight potential size effects and microstructural modulation of strength evolution.

It is acknowledged that the evaluation in this study is based on a single integrated dataset. Although the SHAP-based interpretation aligns with known physical mechanisms, the physical validity of the model remains subject to further verification on external datasets. Future research will therefore focus on extending validation to independent samples and real-world applications to enhance the model’s robustness and credibility. In addition, the proposed framework will be applied to a broader range of rock types and dynamic loading conditions, including multi-axial and cyclic environments. Incorporating microstructural image features and fracture surface descriptors via computer vision techniques holds further promise for improving both predictive accuracy and mechanistic interpretability.

## Figures and Tables

**Table 1:** Statistical description and distribution characteristics of variables.

Index	Specimen Length (mm)	Specimen Diameter (mm)	Grain Size (mm)	Bulk Density (kg/m^3^)	P-Wave Velocity (m/s)	Strain Rate (s^−1^)	Static Strength (MPa)	Dynamic Strength (MPa)
Mean	41.13	42.51	0.77	2499.53	3605.10	60.54	92.27	132.84
Std	15.34	17.50	1.03	192.66	1230.45	51.80	55.43	80.89
Min	10.00	2.50	0.03	2278.00	2437.00	5.00 × 10^−6^	28.60	30.03
1st quartile	34.83	32.00	0.10	2300.00	2812.00	22.40	46.99	68.35
Median	49.78	49.73	0.23	2405.00	3165.00	53.60	71.91	109.00
3rd quartile	50.07	50.11	0.86	2648.00	3955.75	89.25	155.00	193.90
Max	70.00	70.00	3.50	2850.00	6651.00	240.00	212.00	358.00

**Table 2:** Optimized hyperparameters and search ranges for machine learning models.

Model	Hyperparameter	Optimization Range	Result
KNN	n_neighbors	(1, 30)	1.0
	p	(1, 2)	1.0
RF	max_depth	(5, 20)	18.0
	max_features	(1, 5)	4.0
	n_estimators	(100, 500)	150.0
GBDT	learning_rate	(0.01, 0.3)	0.018
	max_depth	(3, 10)	9.0
	n_estimators	(100, 1000)	739.0
LGBM	learning_rate	(0.01, 0.3)	0.28
	max_depth	(3, 10)	4.0
	n_estimators	(50, 500)	450.0
XGBoost	learning_rate	(0.01, 0.3)	0.119
	max_depth	(3, 10)	9.0
	n_estimators	(100, 1000)	364.0
MLPNN	activation	(relu, tanh)	Relu
	alpha	(1 × 10^−5^, 1 × 10^−2^)	0.0057

Note: n_neighbors: number of nearest neighbors used for regression in KNN; p: power parameter for the Minkowski distance metric; max_depth: maximum depth of each decision tree; max_features: number of features considered when splitting a node; n_estimators: total number of boosting iterations or decision trees; learning_rate: step size controlling each tree’s contribution to the final prediction; activation: activation function used in the hidden layer of the neural network; and alpha: L2 regularization parameter (weight decay) in MLP training.

## Data Availability

The original contributions presented in this study are included in the article. Further inquiries can be directed to the corresponding author.

## References

[1] Qian Q., Qi C., Wang M. (2009). Dynamic Strength of Rocks and Physical Nature of Rock Strength. J. Rock Mech. Geotech. Eng..

[2] Cai X., Yuan J.F., Zhou Z.L., Wang Y.M., Chen L.Y., Liu Y., Wang S.F. (2024). Strain Rate-Dependency of Thermal Infrared Radiation of Sandstone Subjected to Dynamic Loading: Insights from a Lab Testing. Int. J. Rock Mech. Min. Sci..

[3] Sun B., Chen R., Ping Y., Zhu Z., Wu N., He Y. (2022). Dynamic Response of Rock-like Materials Based on SHPB Pulse Waveform Characteristics. Materials.

[4] Cai X., Zhou Z., Zang H., Song Z. (2020). Water Saturation Effects on Dynamic Behavior and Microstructure Damage of Sandstone: Phenomena and Mechanisms. Eng. Geol..

[5] Dong L., Wang J., Li X., Peng K. (2018). Dynamic Stability Analysis of Rockmass: A Review. Adv. Civ. Eng..

[6] Deng Z., Liu X., Liu Y., Liu S., Han Y., Liu J., Tu Y. (2020). Model Test and Numerical Simulation on the Dynamic Stability of the Bedding Rock Slope under Frequent Microseisms. Earthq. Eng. Eng. Vib..

[7] Whittington W.R., Oppedal A.L., Francis D.K., Horstemeyer M.F. (2015). A Novel Intermediate Strain Rate Testing Device: The Serpentine Transmitted Bar. Int. J. Impact Eng..

[8] Li W., Tan Z.Y. (2017). Research on Rock Strength Prediction Based on Least Squares Support Vector Machine. Geotech. Geol. Eng..

[9] Wang Y., Hasanipanah M., Rashid A.S.A., Tahir M., Iqbal M., Armaghani D.J. (2023). Advanced Tree-Based Techniques for Predicting Unconfined Compressive Strength of Rock Material Employing Non-Destructive and Petrographic Tests. Materials.

[10] Azarafza M., Hajialilue Bonab M., Derakhshani R. (2022). A Deep Learning Method for the Prediction of the Index Mechanical Properties and Strength Parameters of Marlstone. Materials.

[11] Cheng Y., He D., Liu H., Wang G. (2025). Shear Strength Parameters Prediction of Rock Materials Using Hybrid Machine Learning Model. Nondestruct. Test. Eval..

[12] Wei M., Meng W., Dai F., Wu W. (2022). Application of Machine Learning in Predicting the Rate-Dependent Compressive Strength of Rocks. J. Rock Mech. Geotech. Eng..

[13] Yang Z., Wu Y., Zhou Y., Tang H., Fu S. (2022). Assessment of Machine Learning Models for the Prediction of Rate-Dependent Compressive Strength of Rocks. Minerals.

[14] Lv Y., Zhang R., Zhang A., Shen Y., Ren L., Xie J., Zhang Z., Zhang Z., An L., Sun J. (2024). Machine Learning Algorithms in Rock Strength Prediction: A Novel Method for Evaluating Dynamic Compressive Strength of Rocks Under Freeze–Thaw Cycles. IOP Conf. Ser. Earth Environ. Sci..

[15] Frew D.J., Forrestal M.J., Chen W. (2001). A Split Hopkinson Pressure Bar Technique to Determine Compressive Stress-Strain Data for Rock Materials. Exp. Mech..

[16] Li X., Lok T., Zhao J. (2005). Dynamic Characteristics of Granite Subjected to Intermediate Loading Rate. Rock Mech. Rock Eng..

[17] Xia K., Nasseri M.H.B., Mohanty B., Lu F., Chen R., Luo S.N. (2008). Effects of Microstructures on Dynamic Compression of Barre Granite. Int. J. Rock Mech. Min. Sci..

[18] Wang Y., Tonon F. (2011). Dynamic Validation of a Discrete Element Code in Modeling Rock Fragmentation. Int. J. Rock Mech. Min. Sci..

[19] Zhang Q., Zhao J. (2013). Determination of Mechanical Properties and Full-Field Strain Measurements of Rock Material under Dynamic Loads. Int. J. Rock Mech. Min. Sci..

[20] Zhou Z., Cai X., Chen L., Cao W., Zhao Y., Xiong C. (2017). Influence of Cyclic Wetting and Drying on Physical and Dynamic Compressive Properties of Sandstone. Eng. Geol..

[21] Zhou Z., Cai X., Li X., Cao W., Du X. (2020). Dynamic Response and Energy Evolution of Sandstone Under Coupled Static–Dynamic Compression: Insights from Experimental Study into Deep Rock Engineering Applications. Rock Mech. Rock Eng..

[22] Weng L., Wu Z., Liu Q. (2020). Dynamic Mechanical Properties of Dry and Water-Saturated Siltstones Under Sub-Zero Temperatures. Rock Mech. Rock Eng..

[23] Yu M., Li S., Sun Q., Wang S. (2021). Influence of Grain Size on the Strain-Rate-Dependent Dynamic Response of Sandstones. Geomech. Geophys. Geo-Energy Geo-Resour..

[24] Wang Y., Wang X., Zhang J., Yang B., Chen J., Zhu W. (2021). Experimental Investigation of the Static and Dynamic Compression Characteristics of Limestone Based on Its Initial Damage. Appl. Sci..

[25] Rae A.S.P., Kenkmann T., Padmanabha V., Poelchau M.H., Schäfer F. (2020). Dynamic Compressive Strength and Fragmentation in Felsic Crystalline Rocks. J. Geophys. Res. Planets.

[26] Jerez J.M., Molina I., García-Laencina P.J., Alba E., Ribelles N., Martín M., Franco L. (2010). Missing Data Imputation Using Statistical and Machine Learning Methods in a Real Breast Cancer Problem. Artif. Intell. Med..

[27] Shrestha N. (2020). Detecting Multicollinearity in Regression Analysis. Am. J. Appl. Math. Stat..

[28] Gutmann M.U., Kleinegesse S., Rhodes B. (2022). Statistical Applications of Contrastive Learning. Behaviormetrika.

[29] Pérez N.P., López M.A.G., Silva A., Ramos I. (2015). Improving the Mann–Whitney Statistical Test for Feature Selection: An Approach in Breast Cancer Diagnosis on Mammography. Artif. Intell. Med..

[30] Kardani N., Zhou A.N., Nazem M., Shen S.L. (2021). Improved Prediction of Slope Stability Using a Hybrid Stacking Ensemble Method Based on Finite Element Analysis and Field Data. J. Rock Mech. Geotech. Eng..

[31] Altman N.S. (1992). An Introduction to Kernel and Nearest-Neighbor. Am. Stat..

[32] Breiman L. (2001). Random Forests. Mach. Learn..

[33] Friedman J.H. (2001). Greedy Function Approximation: A Gradient Boosting Machine. Ann. Stat..

[34] Ke G., Meng Q., Finley T., Wang T., Chen W., Ma W., Ye Q., Liu T.Y. LightGBM: A Highly Efficient Gradient Boosting Decision Tree. Proceedings of the 31st International Conference on Neural Information Processing Systems (NeurIPS).

[35] Chen T., Guestrin C. XGBoost: A Scalable Tree Boosting System. Proceedings of the 22nd ACM SIGKDD International Conference on Knowledge Discovery and Data Mining.

[36] Rumelhart D.E., Hinton G.E., Williams R.J. (1986). Learning Representations by Back-Propagating Errors. Nature.

[37] Chicco D., Warrens M.J., Jurman G. (2021). The Coefficient of Determination R-Squared Is More Informative than SMAPE, MAE, MAPE, MSE and RMSE in Regression Analysis Evaluation. PeerJ Comput. Sci..

[38] Futagami K., Fukazawa Y., Kapoor N., Kito T. (2021). Pairwise Acquisition Prediction with SHAP Value Interpretation. J. Financ. Data Sci..

